# A Practical Treatise on the Diseases, Injuries and Malformations of the Urinary Bladder, the Prostate Gland and Urethra

**Published:** 1855-06

**Authors:** 


Art. II.—*A Practical Treatise on the Diseases, Injuries and Malformations of the Urinary Bladder, the Prostate Gland and Urethra.* By S. D. Gross, M. D., Professor of Surgery in the University of Louisville; author of "Elements of Pathological Anatomy;" "A Treatise on Foreign Bodies in the Air-passages," etc., etc. Second edition, revised and much enlarged. With one hundred and eighty-four illustrations. Philadelphia: Blanchard & Lea. 1855. 8vo. pp. 925.

The first edition of this work made its appearance in 1851. Within four years this edition has been exhausted and a second called for. We have it before us. From being unusually well grown at its debut this, the crowning work of Dr. Gross' labors as an author, has acquired a size that must be quite appalling to those who esteem a large book a great evil. And yet, in view of the importance of the subjects discussed, and the almost incredible number of facts which the indefatigable author has collected, its dimensions could hardly have been less. The labors of the reviewer, which if conscientiously performed, are any thing but light,—are greatly sweetened by having the verdict which he pronounces upon new candidates for public favor sustained by his editorial confreres and the bulk of the profession at large. In a review of the former edition of Dr. Gross' work, written for this journal and which, we may remark, was the first analytical notice of it which appeared, we recommended it to our readers as a monograph unequalled, in interest and practical value, by any other on the subject in our language. Subsequent and repeated reference to its pages has confirmed the justness of the opinion we then expressed. About the same time a leading English medical periodical—a source from which American authors are not accustomed to receive much praise—predicted that it would have a "permanent place in the literature of Surgery, worthy to rank with the best works of the present age." It is needless to add that this prediction has been fully verified. The present edition has been augmented by upwards of two hundred pages, and by seventy-eight illustrations.

One of the most interesting and valuable additions is a chapter on the prevalence of calculous disorders in the United States and Canada, which constitutes the first attempt that has ever been made to collect and systematize our information upon the subject. Dr. Gross thinks "there is no reason why the causes of urinary calculi should not be eventually detected, and their formation counteracted, if not prevented, by the timely interposition of remedies, without the necessity of an ultimate resort to the knife." We have no room for extracts—nor are any needed. Most readers in the profession, in the United States, are familiar with the style of our author. We simply reiterate the recommendation we made three years and a half ago. It is unnecessary to do more. D. W. Y.

